# GR-FET application for high-frequency detection device

**DOI:** 10.1186/1556-276X-8-22

**Published:** 2013-01-10

**Authors:** Akram M Mahjoub, Alec Nicol, Takuto Abe, Takahiro Ouchi, Yuhei Iso, Michio Kida, Noboyuki Aoki, Katsuhiko Miyamoto, Takashige Omatsu, Jonathan P Bird, David K Ferry, Koji Ishibashi, Yuichi Ochiai

**Affiliations:** 1 Graduate School of Advanced Integration Science, Chiba University, Chiba, 263-8522, Japan; 2 Department of Chemistry, University of Minnesota Twin Cities, Minneapolis, MN, 55455-0431, USA; 3 Department of Electrical Engineering, University at Buffalo, The State University of New York, Buffalo, NY, 14260-1920, USA; 4 Department of Electrical Engineering and Center for Solid State Electronics Research, Arizona State University, Tempe, AZ, 85287-5706, USA; 5 Advanced Device Laboratory, The Institute of Physical and Chemical Research (RIKEN), Wako, Saitama, 351-0198, Japan

**Keywords:** Graphene, Microwave application, Terahertz detection, Frequency response, Bolometric effect, Nonlinear effect, Ambient condition

## Abstract

A small forbidden gap matched to low-energy photons (meV) and a quasi-Dirac electron system are both definitive characteristics of bilayer graphene (GR) that has gained it considerable interest in realizing a broadly tunable sensor for application in the microwave region around gigahertz (GHz) and terahertz (THz) regimes. In this work, a systematic study is presented which explores the GHz/THz detection limit of both bilayer and single-layer graphene field-effect transistor (GR-FET) devices. Several major improvements to the wiring setup, insulation architecture, graphite source, and bolometric heating of the GR-FET sensor were made in order to extend microwave photoresponse past previous reports of 40 GHz and to further improve THz detection.

## Background

Graphene (GR) has become one of the most well-known carbon nanomaterials due to its unique optical, electrical, and thermal properties which arise from its unique 2D hexagonal honeycomb crystal structure. This unique structure directly influences the band structure of graphene, which is governed by a quasi-Dirac electron system that varies from the single-layer case with gapless spectrum characteristics to the bilayer case with a small forbidden gap [1,2]. In both instances, the band gap can be ideally tuned in order to match the low-energy photons in the gigahertz (GHz)/terahertz (THz) regime. This is in marked contrast to conventional semiconductors whose band gaps appear several orders of magnitude larger. For these reasons, graphene field-effect transistors (GR-FETs) have the potential to exceed the detection limit of most existing semiconductor quantum point contacts [3,4]. This is due to the unique phase-coherent length of open quantum dot structures that can be formed in bilayer graphene when exposed to GHz/THz radiation [5]. An additional benefit of the GR-FET platform in relation to structures based on carbon nanotubes includes the high level of similarity with conventional integrated semiconductor FET fabrication techniques. Considering the mentioned benefits, GR-FETs are emerging as excellent candidates for developing a broadly tunable GHz/THz sensor. In particular, the realization of THz detection will be important for future developments in medical imaging, spectroscopy, and communication, which all exploit the unique linear nonionizing benefits of THz radiation [6].

Existing GR-FETs have been fabricated by micromechanical exfoliation of highly oriented pyrolytic graphite (HOPG-SG2) contacted with two-terminal submicron-scale metal electrodes (Ti/Au or Pd/Au) [5]. The microwave transconductance characteristics show excellent photoresponse around the X band (approximately 10 GHz) but quickly cut off thereafter. The observed cutoff frequency was determined to be a result of the measurement wiring rather than the intrinsic response of the graphene. The positive results of this study indicate that THz detection is possible and that many of the same experimental components could remain constant for THz irradiation experiments. Hence, this study presents the results of such THz irradiation experiment, where the same sample box design used in the previous GHz response measurement was used to test the THz detection capabilities of several GR-FETs. The results of this study and of the former GHz response study revealed numerous complementary areas for improvement. Therefore, this work also investigates experimental improvements to the wiring setup, insulation architecture, graphite source, and bolometric heating detection of the GR-FET sensor in order to extend microwave photoresponse past previous reports of 40 GHz and to further improve THz detection.

## Methods

**Figure 1:**
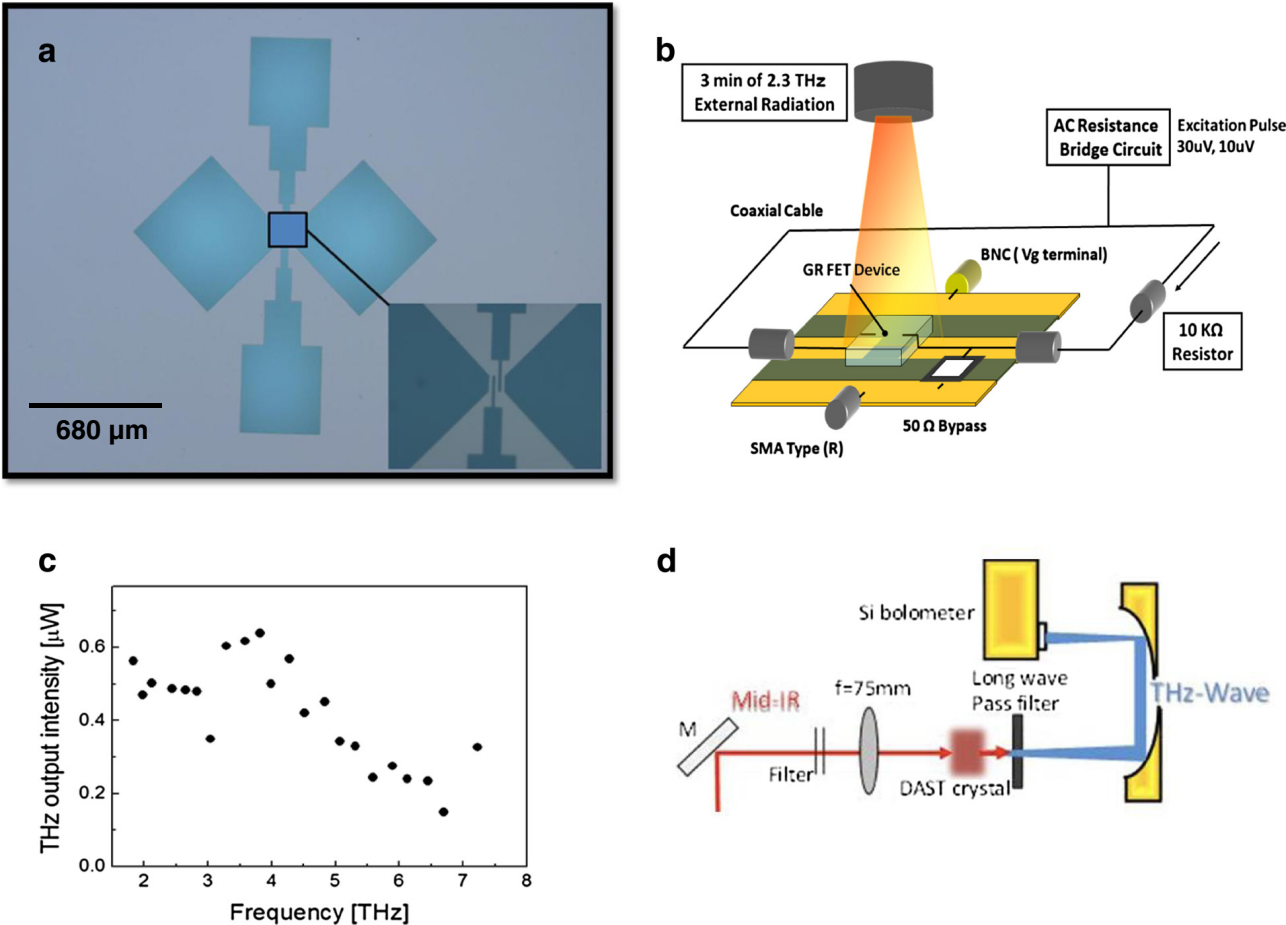
**Experimental overview for THz exposure. **(**a**) Semi-bowtie antenna structure with 680-μm gap dimension custom designed for low THz radiation. (**b**) THz irradiation experimental layout. (**c**) THz wave characteristics at the source-end side of generation. (**d**) THz generation setup.

The devices used in this experiment were fabricated following an established procedure [7]. Thin graphite flakes were exfoliated from natural Kish graphite using adhesive tape and then transferred onto a conducting p-type Si substrate capped with a layer of 300-nm-thick SiO_2_. For comparison purposes, several graphene samples were grown via chemical vapor deposition (CVD) to investigate sample domain size dependence on bolometric sensing performance. Thermal annealing (400°C, flow rate of 4.1 L/min using Ar/H_2_, 5 min) was necessary to remove residual tape adhesive and ambient molecules from the Si substrate surface. The thin graphite flakes were imaged under an optical microscope. Single- and bilayer flakes were identified by examining the light intensity shift in the green channel of the red-green-blue scale relative to the contiguous substrate [8]. Photolithography was performed to form a submicron-scale Ti/Au (50:100 nm thick, respectively) semi-bowtie structure contacts with a 680-μm base separation as depicted in Figure 1a. Overall, three samples were fabricated for THz investigation: sample 2 (bilayer GR), sample 3 (single-layer GR), and sample 4 (single-layer GR grown by CVD). Based on the excellent GHz response previously reported [5], the THz detection capabilities were subsequently investigated. The devices were mounted on a sample box designed to monitor the direct current (DC) characteristics completely insulated from the surrounding noise. The set is portrayed in Figure 1b and was modified to observe the small changes in the DC resistance.

**Figure 2:**
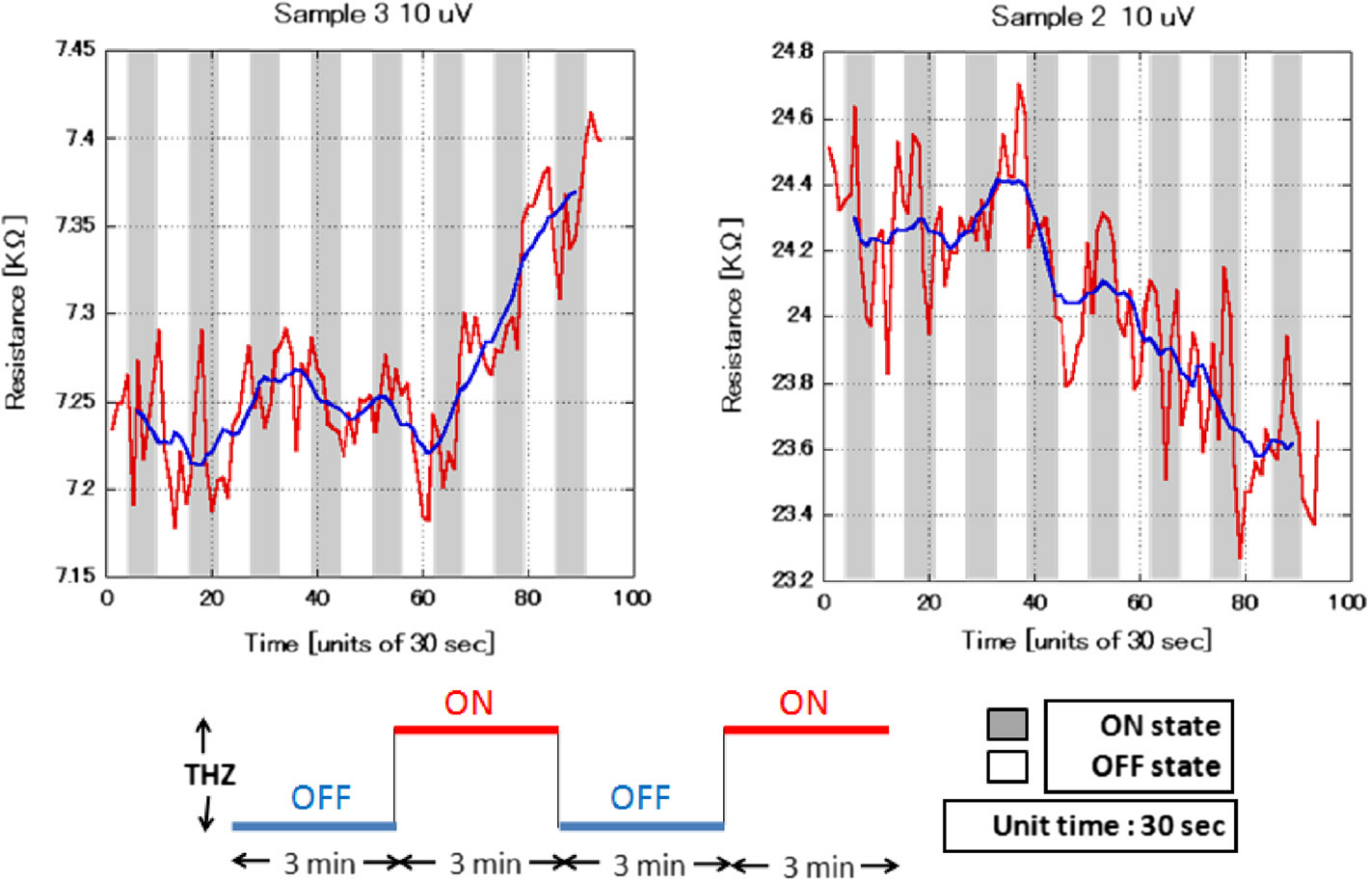
**THz response for sample 2 and sample 3. **The blue line shows the background change which represents the transition in the response modes for the devices, while the red line shows the actual resistance fluctuations due to the THz radiation.

THz exposure pattern followed transition sequences between THz-ON/THz-OFF states for periods of 3 min as seen in Figure 2. The THz power was estimated to be 500 nW at the source-end as in Figure 1c[9].

**Figure 3:**
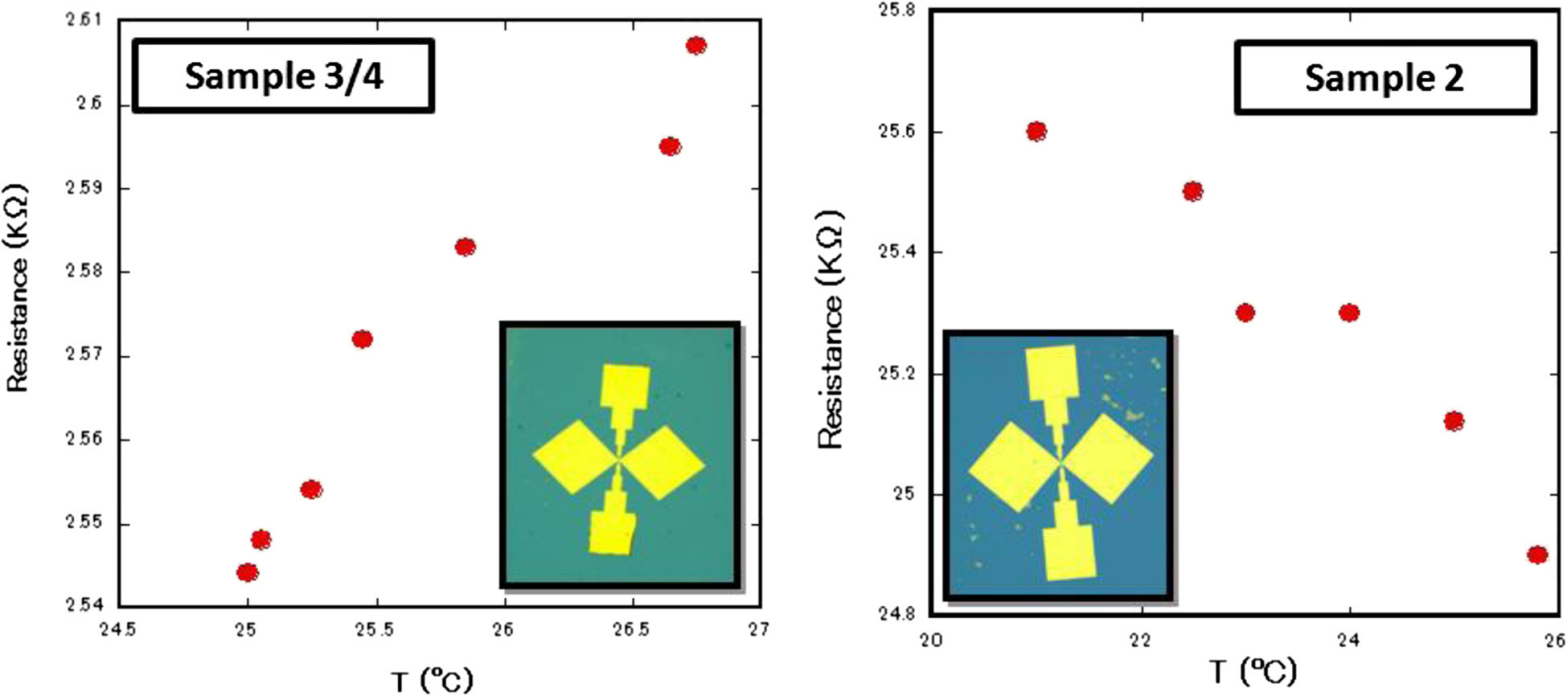
**Sample resistance change due to temperature variation around room temperature. **The left graph shows a metallic response from samples 3 and 4 (monolayer GR device). The right graph shows a semiconductor response from sample 2 (bilayer GR device). The two devices shown as insets are implemented using the mask patterns of Figure 1a. They are identical except for the graphene thickness.

The change in the resistance was recorded every 30 s. Finally, the change in the sample resistance as a function of temperature was confirmed in accordance with the graphene layer thickness as shown in Figure 3. The associated characteristics of each device type, monolayer being semimetallic and bilayer being semiconducting, were used to explain the relative response to THz radiation as bolometric response.

**Figure 4:**
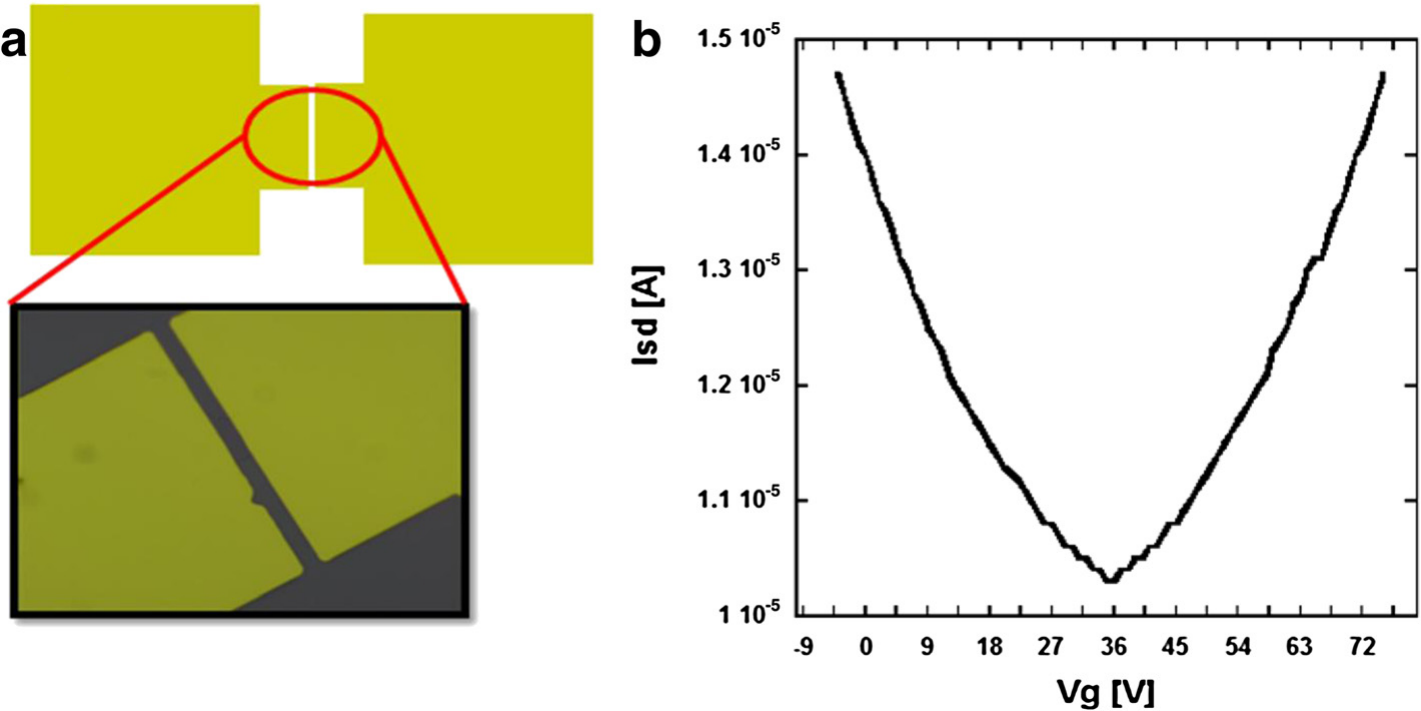
**Characteristics for a GR-FET GHz detector. **(**a**) Basic two-terminal metal contact. (**b**) Gate voltage dependence for a bilayer GR-FET at room temperature with observable Dirac point.

Furthermore, in our recent attempt to improve the microwave transport characteristics, a new setup was used to improve the response of high-frequency operation modes. A simple two-terminal Ti/Au (50:100 nm thick, respectively) design with a gap of 10 μm was used for the GHz response experiment as seen in Figure 4a. Back-gate dependence measurements were conducted at room temperature by varying the DC two-terminal current as a function of the gate voltage. The Dirac point or minimum conductivity point was located around 35 V as seen in Figure 4b. GHz frequency response measurements were taken up to 40 GHz at zero back-gate voltage using an improved experimental setup. Structural changes are highlighted in the discussion later on. The device is supported by a back-gate voltage platform and connected to the 40-GHz signal generator and power sensor through a combination of Cu/Au wires after passing through subminiature type K (SMK) connectors.

## Results and discussion

Based on our previous discussion of the microwave transport properties in GR-FET devices [5], the possibility to utilize GR for THz detection has become a more practical goal. Following the previously discussed approach, a clear response to THz radiation has been observed using the setup shown in Figure 2. The fluctuations in the response of the device can be explained by considering the influence of bolometric and nonlinearity effects within the GR material. Exposure to THz radiation will inevitably induce these effects depending on the nature of the sample, whether it is monolayer with semimetallic behavior or bilayer with semiconductor behavior, resulting in a change in the resistance. Referring back to the original resistance's room temperature dependence in Figure 3, the outcome of Figure 2 can be understood to be the result of a strong bolometric response that increases the resistance in the metallic-type devices and decreases the resistance in the semiconductor-type devices. In addition, nonlinearity effects play an important role in influencing the response of semiconductor-type devices to THz radiation. Nonlinear response occurs because the band gap excitation energy matches the incident wave frequency.

**Figure 5:**
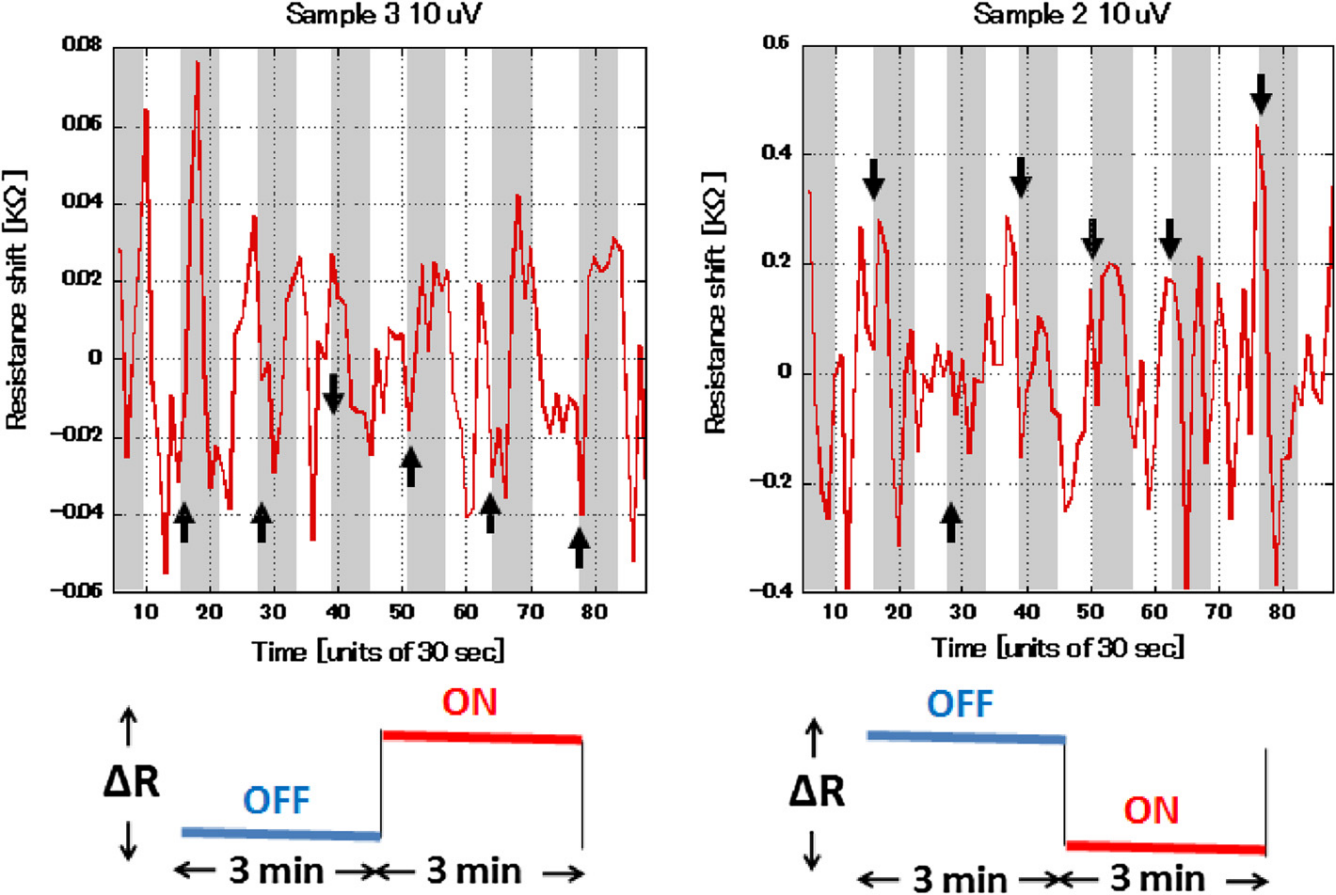
**Resistance fluctuation and amplitude response for THz irradiation. **Most cycles follow a bolometric response, while a few cases follow a nonlinear response. The nonlinear response arises from the excitation of extra carriers which is reflected as an opposite response in the resistance change compared to the bolometric response.

Transitions between THz ON and OFF exposure states change the resistance values in a manner that can be explained by bolometric and nonlinearity effects for both monolayer and bilayer devices. The flat regions of the curves within the first four cycles for sample 3 and the first three cycles for sample 2 show the transitions in the responses between the expected bolometric response and occasionally the nonlinear response. After a short period of time, the response is completely dominated by bolometric effects. To clarify the real bolometric impact, the blue background is subtracted to show the absolute resistance change. Fluctuation amplitude can be clearly seen in Figure 5[10,11]. The observed results show a clear distinction between the response of single- and bilayer devices in sensing THz radiation.

The main aspects of characterization were indicated by the small arrows in the previous response curves of Figure 5; the arrows simply indicate two sets of information. The first aspect is the change in the average resistance value for the transition from the THz-OFF state to the THz-ON state. The second aspect is the instantaneous value of the resistance at the two moments where THz radiation starts and the moment where THz radiation is terminated.

**Figure 6:**
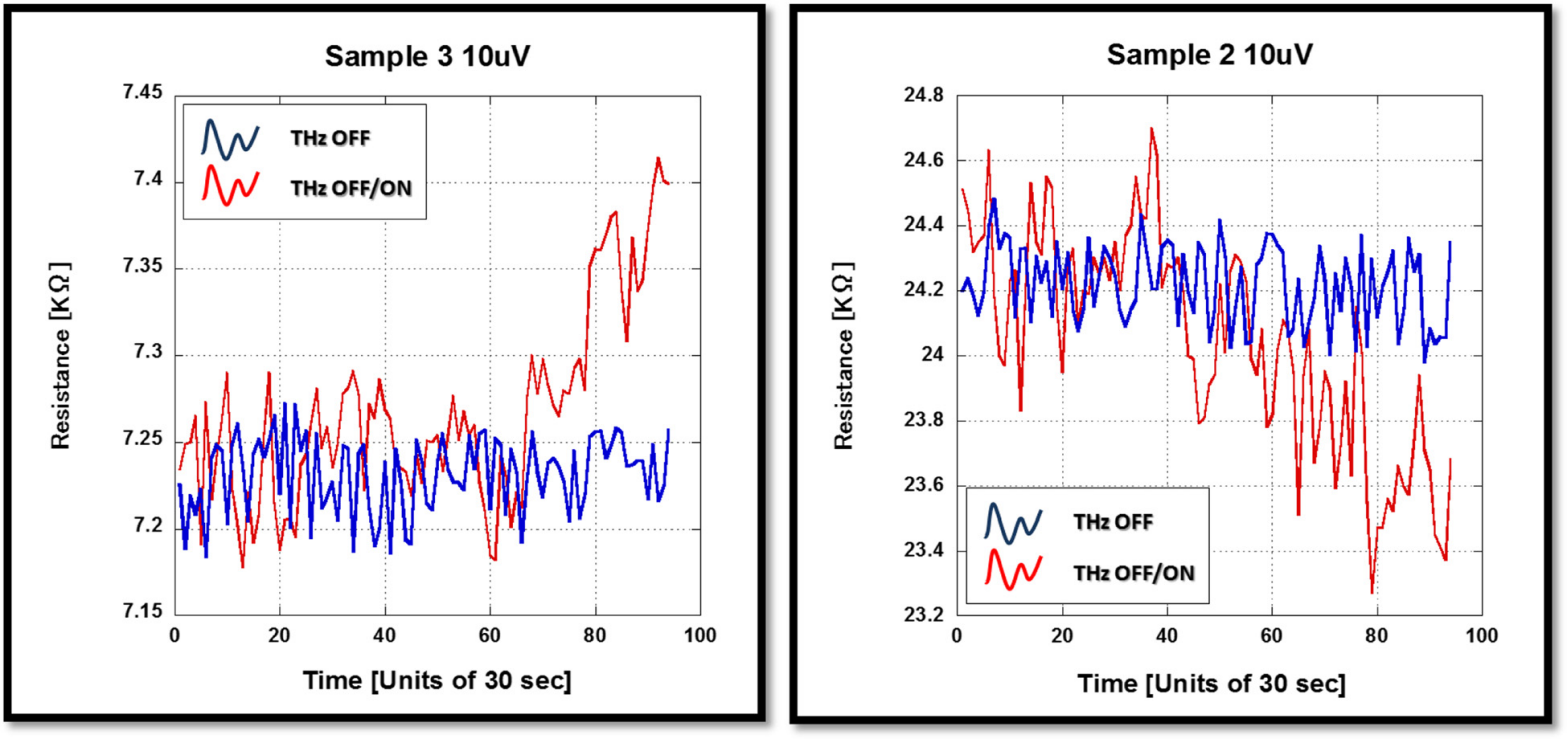
**Comparison of the resistance response between THz OFF-ON states and the complete THz-OFF state. **The THz-OFF measurement was taken for 10 min and plotted as the blue curve. The same measurement is also fitted on the OFF-ON state measurement to indicate the variation of the fluctuation amplitudes. The background of the plot variation can be viewed as a result of room temperature dependence.

Furthermore, looking into the data analysis, sample 3 (metallic type) and sample 2 (semiconductor type) started in the THz-OFF state for 3 min where the average fluctuation amplitude was estimated to be 0.03 and 0.15 KΩ, respectively. Pulsed THz radiation was applied for 3-min intervals, as indicated by the gray-shaded regions in Figure 2. The devices' bolometric response to THz radiation is reflected by the correlating resistance amplitude fluctuations. Examining Figure 6, the differences in fluctuation amplitudes show a clear variation between complete THz-OFF and THz OFF-ON states. Metallic characteristics are observed for sample 3 after three successive cycles of exposure with an amplitude increase of 0.05 KΩ. Conversely, sample 2 shows semiconductor characteristics after two successive cycles of exposure with an amplitude decrease of 0.40 KΩ. The fluctuation amplitudes increase by a factor of 2 relative to the original THz-OFF state. Cycle 4 for sample 3 and cycle 3 for sample 2 show opposite responses since the change due to THz-ON radiation does not fade out with the THz-OFF state. Consequently, the response shows a linear growth for the fluctuation amplitudes. The metallic sample's average fluctuation amplitude increases by 0.08 KΩ during the THz-ON state, while the semiconductor sample's average fluctuation amplitude decreases by 0.65 KΩ during the THz-ON state. The fluctuation amplitudes changed by a factor of 3 relative to the original THz-OFF state. These trends can be observed in comparison to the original fluctuation as shown in Figures 5 and 6. Transitions in response occur in correspondence to the opposite response observed in cycle 4 of sample 3 and cycle 3 of sample 2, as shown in Figure 5.

**Figure 7:**
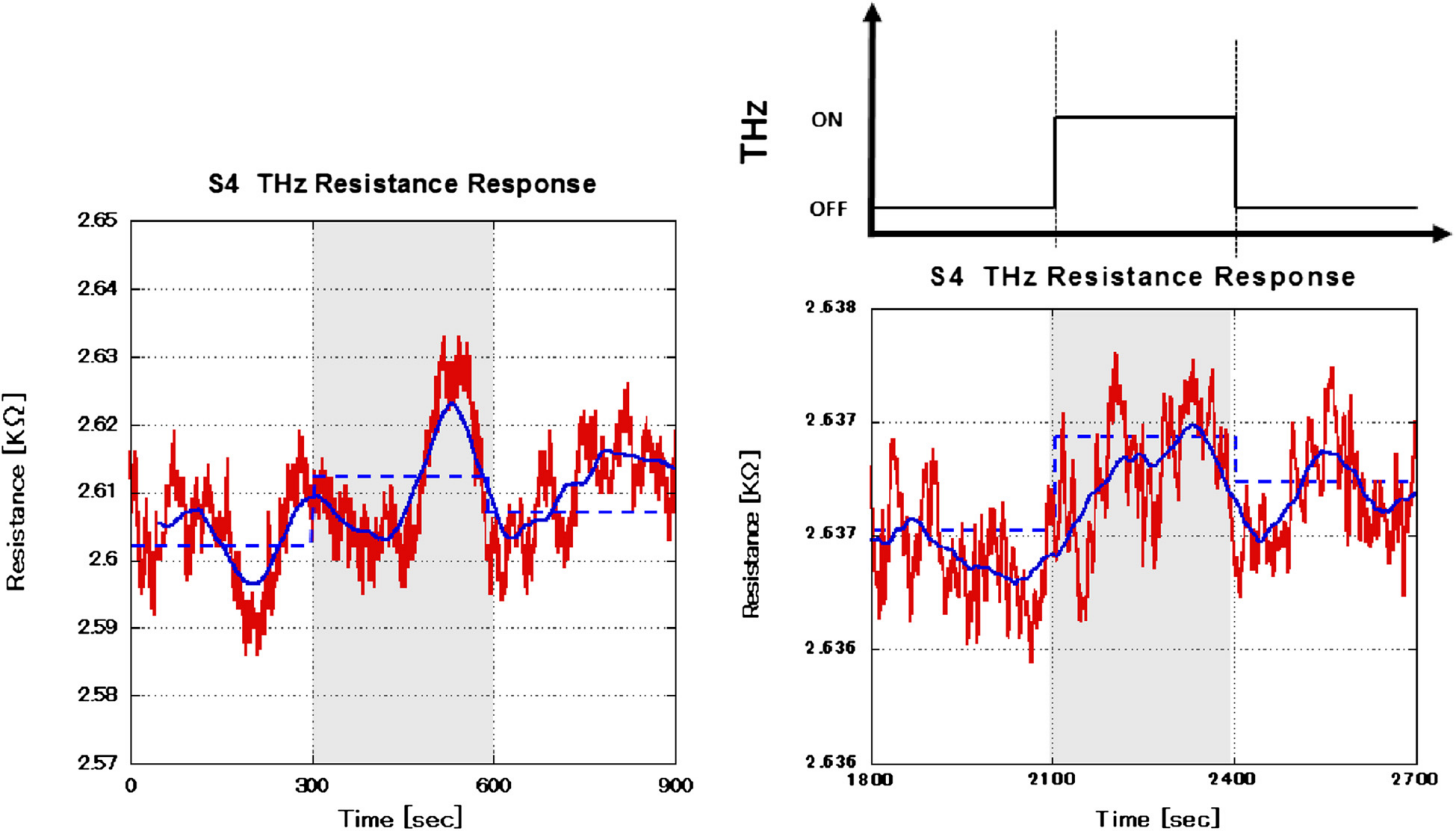
**Response of sample 4 (CVD, monolayer GR) to THz radiation. **Due to a large sample size domain of 10 mm^2^, higher thermal energy is required to induce a sufficient bolometric response. The red solid line shows the actual data. The blue solid line shows the background change which represents the transition in the response modes for the device. The blue dashed line shows the average value of the resistance. The two figures correspond to two different time segments to imply the response regeneration.

Finally, the efficiency of inducing the thermal energy required to observe a bolometric response has been related to the sample's domain size at the core of the antenna structure. Figure 7 shows the response of sample 4, which was obtained by CVD. The domain size of sample 4 is 10 mm^2^ and is 4 orders of magnitude larger than that of the exfoliated samples. Following a similar approach as described previously, the sample started in the THz-OFF state for 5 min where the average fluctuation amplitude was estimated to be 10 Ω. The tendency for bolometric response is reflected by the observed fluctuation amplitudes of the resistance. The differences in fluctuation amplitudes show the variation between complete OFF and ON states. Sample 4 shows a metallic characteristic with a fluctuation amplitude of 20 Ω, which reflects an increase by a factor of 2 relative to the original THz-OFF state.

Overall, this experiment reveals the interplay of different photoresponse mechanisms primarily involving rectification due to THz radiation in the presence of nonlinearity and bolometric heating effects on the transport properties of GR-FET devices. The observation of such bolometric responses, especially at ultrahigh frequencies, is a highly prized characteristic for a variety of device applications. Similarly, such a response has been observed for GaAs [4], which confirms the bolometric behavior observed in the GR-FET device, even at ambient conditions.

**Figure 8:**
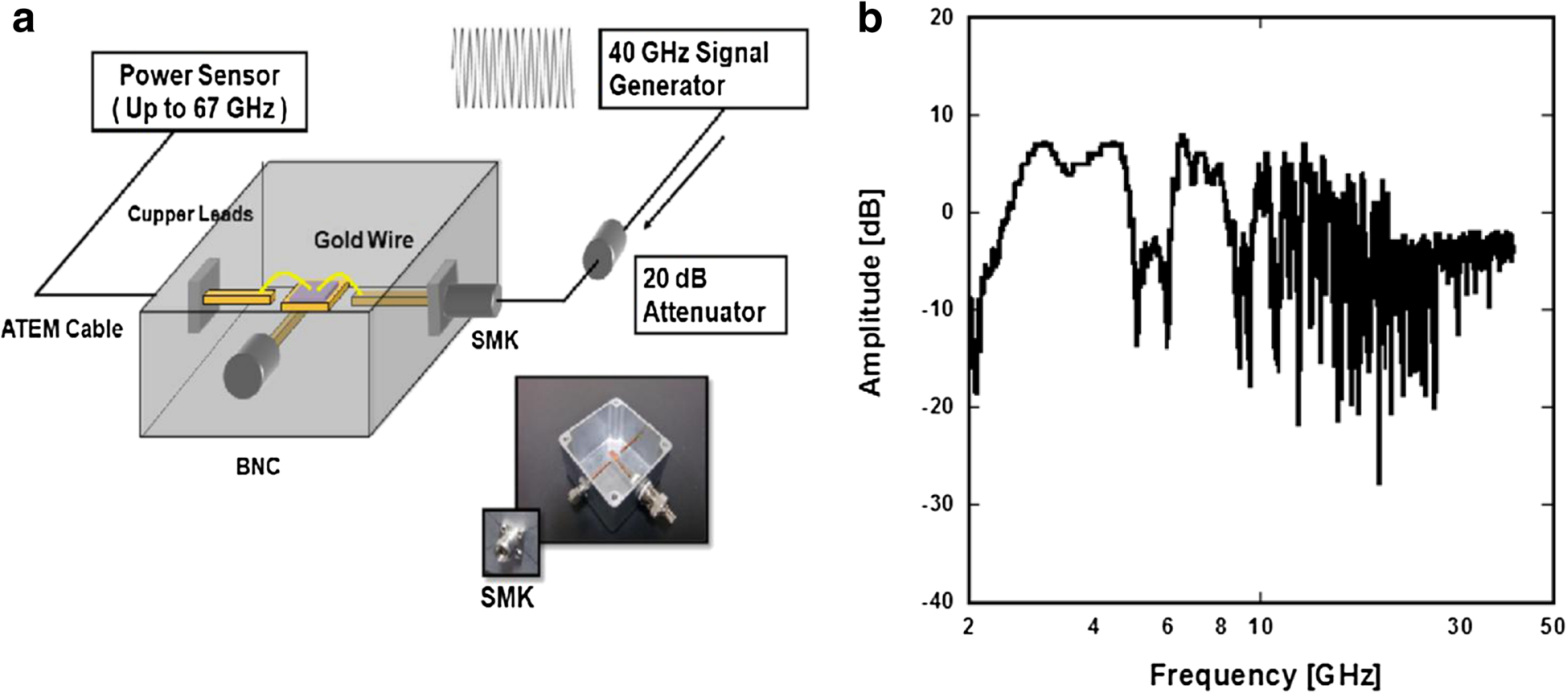
**The GHz transmission setup. **(**a**) Modified box with floating lead connections that have drastically reduced the surface resonance effects and parasitic capacitance. (**b**) Frequency response profile for the transmitted signal up to 40 GHz.

Realizing the need to improve our measurement setup, several modifications to the sample box shown in Figure 8a were made in order to extend the detection limit of our device. Modifications, such as suspending the device using Cu/Au wires rather than having it rest on an insulating substrate, were found to greatly reduce parasitic capacitance and increase the detection limit of the device. As discussed previously [5], using SMA connectors presented a major limitation in the previous setup and affected the total response cutoff. In our recent attempt, SMK connectors and cables were used which have a higher cutoff frequency at 40 GHz. Therefore, the device response was predominantly limited by surface wave resonance effects from the metal plate stage and the lead contacts as demonstrated in Figure 8a. The device response shows possible conduction modes for the GR device up to 50 GHz, indicating that the ‘yield’ has drastically increased. At higher frequency regimes, a greater gain in amplitude relative to the starting point is observed, showing that the transport modes dominate the device performance as shown in Figure 8b.

## Conclusions

The observation of a high-frequency response in GR-FETs beyond 40 GHz has clarified the importance of power and intensity in microwave transmission. Following a previous study in semiconductor QD THz sensing [4], a basic frequency characteristic has already been defined using a conventional microwave transconductance measurement [5]. Building on these findings, this experiment presents a systematic study which explored the GHz/THz detection limit of both bilayer and single-layer GR-FETs. THz irradiation experiments revealed the interplay of different photoresponse mechanisms, primarily involving nonlinearity and bolometric heating effects on the transport properties of the GR-FET device. The bilayer GR samples show a clear visible - faster and larger - photoresponse change in comparison to the monolayer sample. This is a direct result of the small apparent band gap that exists in the bilayer GR materials. The observation of such bolometric responses, especially at ultrahigh frequencies, is a highly prized characteristic for a variety of device applications. Additionally, the microwave response of both the single- and bilayer GR-FET was significantly extended from previous reports by improving the wiring setup, insulation architecture, and heat dissipation of the GR-FET nanosensor. Even in the case of the GR two-terminal system, an excellent response was observed under room-temperature conditions [5]. Therefore, it is possible to conclude that the GR strip line detector system serves as a valuable means to analyze high-frequency response measurements and that GR-FETs will work effectively as room-temperature GHz-THz sensors.

## Abbreviations

DC: Direct current; GHz: Gigahertz; GR: Graphene; GR-FET: Graphene field-effect transistor; QD: Quantum dot; THz: Terahertz.

## Competing interests

The authors declare that they have no competing interests.

## Authors’ contributions

YO conceived the main idea. AMM and AN developed the approach and carried out the main sample preparation, experimental process, and data interpretation. TA, YI, and TO aided on the data analysis and helped on the terahertz experiment. MK helped effortlessly on the experimental setup. KM and TO mainly provided the required setup for the terahertz radiation and provided a long-time collaboration with our laboratory. NA, JPB, DKF, and KI mainly worked on the theoretical background of the study. All the authors contributed to the preparation and revision of the manuscript, and read and approved the final manuscript.

## Authors’ information

YO is a regent professor; NA is an associate professor; AMM, TA, YI, and TO are graduate students; MK is a postdoctoral candidate; TO is a professor; and KM is an assistant professor from the Graduate School of Advanced Integrated Science at Chiba University. AN is an undergraduate student from the Chemistry Department at the University of Minnesota-Twin Cities. JPB is a professor in the Electrical Engineering Department, SUNY at Buffalo. DKF is a regent professor in the Department of Electrical Engineering, Arizona State University. KI is a professor in the Advanced Device Laboratory at the Institute of Physical and Chemical Research (RIKEN).
